# In sickness and in health: the dynamics of the fruit bat gut microbiota under a bacterial antigen challenge and its association with the immune response

**DOI:** 10.3389/fimmu.2023.1152107

**Published:** 2023-04-11

**Authors:** Tali S. Berman, Maya Weinberg, Kelsey R. Moreno, Gábor Á. Czirják, Yossi Yovel

**Affiliations:** ^1^ Department of Zoology, Tel Aviv University, Tel Aviv – Yafo, Israel; ^2^ Department of Wildlife Diseases, Leibniz Institute for Zoo and Wildlife Research, Berlin, Germany; ^3^ Sagol School of Neuroscience, Tel Aviv University, Tel Aviv – Yafo, Israel

**Keywords:** immune response, Chiroptera, ecoimmunology, *Weissella*, gut microbiota resilience, 16s RNA, SILVA database, host-pathogen interactions

## Abstract

**Introduction:**

Interactions between the gut microbiome (GM) and the immune system influence host health and fitness. However, few studies have investigated this link and GM dynamics during disease in wild species. Bats (Mammalia: Chiroptera) have an exceptional ability to cope with intracellular pathogens and a unique GM adapted to powered flight. Yet, the contribution of the GM to bat health, especially immunity, or how it is affected by disease, remains unknown.

**Methods:**

Here, we examined the dynamics of the Egyptian fruit bats’ (*Rousettus aegyptiacus*) GM during health and disease. We provoked an inflammatory response in bats using lipopolysaccharides (LPS), an endotoxin of Gram-negative bacteria. We then measured the inflammatory marker haptoglobin, a major acute phase protein in bats, and analyzed the GM (anal swabs) of control and challenged bats using high-throughput 16S rRNA sequencing, before the challenge, 24h and 48h post challenge.

**Results:**

We revealed that the antigen challenge causes a shift in the composition of the bat GM (*e.g., Weissella, Escherichia, Streptococcus*). This shift was significantly correlated with haptoglobin concentration, but more strongly with sampling time. Eleven bacterial sequences were correlated with haptoglobin concentration and nine were found to be potential predictors of the strength of the immune response, and implicit of infection severity, notably *Weissella* and *Escherichia*. The bat GM showed high resilience, regaining the colony’s group GM composition rapidly, as bats resumed foraging and social activities.

**Conclusion:**

Our results demonstrate a tight link between bat immune response and changes in their GM, and emphasize the importance of integrating microbial ecology in ecoimmunological studies of wild species. The resilience of the GM may provide this species with an adaptive advantage to cope with infections and maintain colony health.

## 1 Introduction

All animals host microbial communities (bacteria, viruses, archaea, fungi, protists and their genomes; *i.e.*, microbiome), which significantly affect their biology and evolution (1–4). The microbiome, particularly the gut microbiome (GM), is known to impact host survival by influencing physiology, nutrition and behavior (gut-brain axis (5);). Furthermore, interactions between the GM and the immune system are essential for maintaining host health (5–7). The GM contributes to the development of the host immune system during early life stages, regulates it to maintain gut homeostasis and protects the host from colonization of potential pathogens while enabling the presence of beneficial microbes (6, 7). At the same time, the host immune system can modify GM composition and diversity, ultimately affecting infection dynamics (8–11). However, despite numerous studies on domestic and laboratory animals, only a few studies have investigated GM dynamics during health and disease in wild species, which are constantly exposed to pathogens and environmental changes (12).

Bats (Chiroptera) are a highly diverse order of mammals which are capable of sustained flight, allowing them to inhabit many ecological niches and environments. As such, they are an ideal model system for studying microbiome ecology and evolution (13). The bat microbiome was found to be essential to processes such as food absorption (14), social recognition (15, 16) and pathogen defense (17). However, studies on microbial communities of bats and their role in bat health are scarce compared to studies on the bat virome (5, 13, 18). Recent studies have shown that the bat GM resembles that of birds (*i.e.*, dominated by Proteobacteria), rather than that of mammals (dominated by Bacteroidetes (5, 16)), and that both internal (sex, genetics and physiological state) and external (environment, season and diet) factors influence the bat GM diversity and composition (16, 19–22). Moreover, the bat fur and gut bacterial communities display significant temporal and spatial shifts, which are also synchronized at the colony level (16, 23). Nonetheless, studies on the associations between disease, immunity and GM variability in bats are scarce (11, 24) and the contributions of the GM to bat health remain largely unknown, despite their recognized role as natural reservoirs of several pathogens (5).

In this study, we experimentally investigated the dynamics of the GM of Egyptian fruit bats (*Rousettus aegyptiacus*) during health and disease, with special focus on its association with innate immunity. Since this frugivorous species lives in large crowded colonies, active transmission of pathogens between individuals is likely common (25, 26), making them good candidates for examining associations between the GM and disease dynamics. We provoked an inflammatory response in bats using lipopolysaccharides (LPS), a component of the outer membrane in Gram-negative bacteria, that induces an acute phase response. LPS has previously been used to induce innate immune responses in *R. aegyptiacus* and other bat species, with species specific outcomes in term of fever, leukocytosis, body weight loss and elevated levels of the acute-phase proteins, haptoglobin and lysozyme (see (27)). The inflammatory marker haptoglobin was measured and the GM of healthy (control) and immune challenged bats (henceforth, “challenged bats”) was analyzed before the administration of LPS and over a period of 48 hours in order to examine: 1) the diversity and structure of the bat GM following an immune challenge, 2) the associations between the immune response and shifts in the GM, 3) whether GM structure prior to the challenge affects the strength of the immune response and which specific bacterial taxa are responsible for this.

## 2 Methods

### 2.1 Study animals

Adult male Egyptian fruit bats (n=26) were captured in June 2020 at a cave colony in Herzliya, Israel. They were examined for ectoparasites and treated topically with carbaryl powder (Opigal, product no. 88000029, Abik, Israel) to reduce parasitic load. After a general health check by an experienced veterinarian, the bats were housed together in an experimental room (245cm × 200cm × 210cm) at 25°C, with a natural dark-light cycle of 12h for four days, in order to acclimate to captivity housing and feeding. Each individual was provided with 150g of diced fruit (banana, apple and melon) daily and uniquely marked for identification using hair bleach on its back.

### 2.2 Experimental setup

#### 2.2.1 Immune challenge

Individuals were randomly assigned to a control (n=6, hereafter ‘control bats’) and a treatment group (n=20, challenged bats). Challenged bats were injected subcutaneously with LPS from *Escherichia coli O111:B4* (L2630, Sigma Aldrich, MO, USA) at a concentration of 2mg/kg bodyweight (0.577 ± 0.144mg) diluted in sterile phosphate-buffered saline (PBS, P5493, Sigma-Aldrich, MO, USA) (28). The experiment (*i.e.*, injection of LPS or PBS) was conducted in two rounds, each consisting of 10 challenged bats and three control bats (total of 13 bats per round). GM and blood samples and body weight measurements were taken for each bat at three time points - pre-injection, 24h and 48h post-injection (**Data Sheet S1**).

#### 2.2.2 Gut microbiome sampling

The GM was sampled by holding each bat and squeezing the anus to extract a transparent discharge. This discharge was swabbed using sterile culture swab applicators (BD CultureSwab™, BD Biosciences, San Jose, CA) moistened with Ringer’s Solution (AWB2324A, Teva Pharmaceutical Industries Ltd., Israeli). *R. aegyptiacus* has a relatively short intestine, with no observed cecum or appendix (29, 30), and food transit through the intestine takes about 40 minutes. Thus, the anal discharge is a good representation of the core GM (16). After sampling, the swabs were sealed in their sterile containers and kept at -80°C until DNA extraction.

#### 2.2.3 Blood collection

Approximately 1,000μl of blood was collected *via* venipuncture with micro container separation gel tubes (BD SST™ Serum Tube with Separating Gel, NJ, USA) from the antebrachial or the wing vein using different locations for each sampling. The blood samples were cooled and centrifuged at 12000×g for three minutes to separate the serum. The serum was then collected and stored at -80°C until further analysis. All individuals were offered mango juice immediately after handling.

### 2.3 Measuring haptoglobin concentration

In previous studies, bats (including *R. aegyptiacus*) that received LPS exhibited elevated levels of the acute-phase protein, haptoglobin (see (27)), suggesting that this protein plays a major role in antibacterial and antifungal defense in bats (31–33). Haptoglobin concentration was measured using the “PHASE” TM Haptoglobin Assay (Tridelta Development, Maynooth, Ireland) according to the manufacturer’s instructions, as done in other bat species (31, 34). Briefly, plasma samples were diluted (1:2) with PBS and hemoglobin was added. The peroxidase activity of hemoglobin, which is preserved by being bound to haptoglobin, is directly proportional to the amount of haptoglobin in the sample. Thus, haptoglobin concentrations (mg/ml) were estimated based on the peroxidase activity of hemoglobin, according to the standard curve on each plate.

### 2.4 Analyses of gut microbiome samples

#### 2.4.1 Molecular analysis and library construction

DNA was extracted from the swabs using the PowerSoil^©^ DNA isolation Kit (MoBio Laboratories, Carlsbad, CA), as recommended by the manufacturer, and stored at −20°C. PCR amplification of the 16S rRNA gene was carried out with universal prokaryotic primers containing 5-end common sequences (CS1-341F 5’-ACACTGACGACATGGTTCTACANNNNCCTACGGGAGGCAGCAG and CS2-806R 5’-TACGGTAGCAGAGACTTGGTCTGGACTACHVGGGTWTCTAAT). A total of 28 PCR cycles were conducted (95°C for 15s., 53°C for 15s, 72°C for 15s) using the PCR mastermix KAPA2G Fast™ (KAPA Biosystems, Sigma-Aldrich). Negative controls were carried out for each PCR assay. Products were verified by agarose gel electrophoresis and stored at -20°C. 16S rRNA amplicon sequencing was performed on an Illumina MiSeq instrument at the Sequencing Core (UICSQC), University of Illinois, Chicago, using a two-stage amplification protocol (35). Sequencing results (pair-ends 2x250 bp) were delivered as FASTQ formatted files.

#### 2.4.2 Sequence analysis and taxonomic identification

The FASTQ files were processed using the DADA2 pipeline (36) in R. Briefly, reads were first trimmed and filtered (command: ‘filterAndTrim’) with the parameters truncLen=c (280,220), maxN=0, maxEE=c(2.2), trimLeft=c (23, 26). Error rate estimation (command: ‘learnerror’) was performed using default parameters, with the randomize parameter set as ‘TRUE’ in order to randomly sample nucleotides and reads for the model building process. Sequencing errors were inferred using the DADA2 algorithm, and forward and reverse reads were merged (command: ‘mergePairs’). Following chimera removal (command: ‘removeBimeraDenovo’), an amplicon sequence variants (ASVs) table was constructed containing 2,649,278 high-quality reads binned in 1,426 ASVs and 78 samples. Taxonomy was assigned using the SILVA 16S ribosomal RNA database implemented in DADA2 (command: ‘assignTaxonomy’; release 138.1 (37);). Sequences which could not be classified (no hit), ASVs assigned to archaea, and those assigned to mitochondria or chloroplasts were removed. Two challenged bats lacking sufficient GM and/or blood sample data for all three time points (pre-injection, 24h and 48h post-injection) were also removed from the analysis, retaining 2,438,779 quality reads binned in 1,235 ASVs and 72 samples (18 challenged and six control bats; **Data Sheet S2**). The raw sequence data is available at the NCBI database under BioProject accession number PRJNA893867.

### 2.5 Data analysis

All statistical analyses were performed in R version 4.1.1. To begin, the different metadata variables (haptoglobin, body weight and sampling time) were tested for collinearity using the ‘rcorr’ function (type = “spearman”) in the ‘Hmisc’ R package (**Figure S1**). Haptoglobin concentration and body weight were compared between control and challenged bats over three sampling times using an Aligned Rank Transform (ART) ANOVA in the ‘ARTool’ R package (38). This non-parametric test was chosen since the haptoglobin data showed no homogeneity of variance (verified by F test) or normal distribution (verified by Shapiro Wilk test).

In order to estimate sampling adequacy, a rarefaction analysis was performed using PAST (39), which indicated sufficient depth of sampling (**Figure S2**). The data was then rarefied to a minimum sequence depth of 20,000 sequences per sample using the command ‘rarefy’ in the VEGAN R package, in order to avoid sequencing depth-relate bias. This resulted in 69 samples (the samples of an additional LPS bat were removed from the analysis as these contained less than 20,000 sequences per sample) clustered in 1,195 ASVs (**Data Sheet S3**). Following, diversity (Shannon) and richness (Fisher) indices were calculated for the ASV’s using the ‘diversity’ command in the VEGAN R package and compared between the treatment groups and sampling times using the non-parametric ART ANOVA test (see above).

The relationship between the GM composition, sampling time and treatment group (beta diversity) was examined using a canonical correspondence analysis (CCA). For this purpose, the non-rarefied read data were normalized using the cumulative sum of squares method (CSS) in the metagenomSeq R package (**Data Sheet S4**). CCA was then performed using the command ‘cca’ in the VEGAN R package, with sampling time and treatment group set as constrained variables, and bat ID set as a conditional variable. A permutation test for CCA (‘anova.cca’) was used to check the significance of the CCA model. To examine the contribution of treatment, sampling time and haptoglobin concentration (and their interactions) to variation in the GM composition, a permutational multivariate analysis of variance (PERMANOVA) was run with 999 permutations using the VEGAN function ADONIS, with bat identity set as a nested parameter. Since sampling time and haptoglobin concentration were co-linear (r=0.64, **Figure S1**), the PERMANOVA test was performed separately, once for treatment and sampling time, and once for treatment and haptoglobin concentration (treatment × sampling time/bat identity; treatment × haptoglobin/bat identity).

To investigate the relationship between the infection and changes in the GM composition, a Spearman correlation test (command ‘corr.test’ in the psych R package) was performed between ASV and haptoglobin concentration of challenged bats. The Benjamini and Yekutieli (“BY”) multiple hypothesis testing correction method was applied to the results.

To examine whether the strength of the immune response (increase in haptoglobin concentration of bats depends on the composition of their GM prior to the infection, we checked which of the ASVs at time 0h (pre-injection of LPS) could be predictive of the haptoglobin concentration 24h after the injection of LPS (when the shift in the GM composition was most evident). To do so, a cutoff level was determined for haptoglobin at time 24h based on the median concentration - low haptoglobin (*i.e.*, mild immune response) = <7mg/ml and high haptoglobin (*i.e.*, strong immune response) = >7mg/ml. The challenged bats (n=18) were then divided into two groups, exhibiting mild or strong immune responses, based on their haptoglobin concentration at time 24h and a linear discriminant analysis (LDA) effect size (LEfSe) was performed. Only prevalent ASVs (>10% in all samples; 241/1,235) were included in this analysis. The LefSE analysis was performed with CSS normalized counts (cumulative sum of squares method, see above) of ASVs before the administration of LPS (time 0h) against the haptoglobin concentration (high or low) 24h after the administration of LPS, using the online tool Galaxy (version 1.0; http://huttenhower.sph.harvard.edu/galaxy/) with default parameters (without the counts per million transformation). The threshold for the logarithmic LDA score for discriminative features chosen was >2.0.

Finally, we performed a linear decomposition model (LDM) using the LDM package in R (40) to test for the effects of treatment and sampling time on the most prevalent ASVs (>60% of samples). Differences in the relative abundance of the ASVs were detected by the LDM, while controlling false discovery rate (FDR) using the Benjamini and Hochberg method (for results see **Data Sheet S5–7**).

## 3 Results

### 3.1 Bats’ physiological response

**Figure 1:**
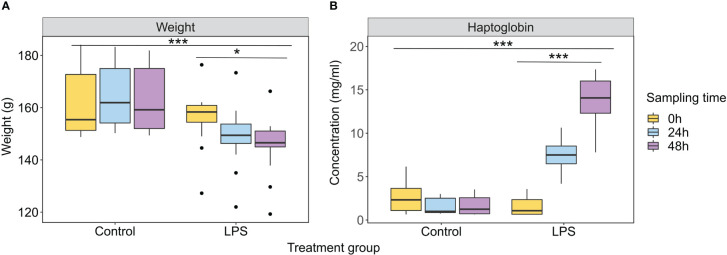
Physiological responses of bats from the control and treatment (LPS) groups during three sampling times. **(A)** Body weight. **(B)** Haptoglobin concentration. Results are presented as mean ± SEM. *p<0.05, ***p<0.001. For full ART ANOVA statistics see **Table S1**.

Challenged bats (*i.e.*, injected with LPS) showed a significant decrease in body weight and an increase in haptoglobin concentration compared to healthy control bats (ART ANOVA: body weight: time × treatment - F_1,44 =_ 9.827, *p*<0.001, **Figure 1A**; haptoglobin: time × treatment - F_1,44 =_ 78.773, *p*<0.001, **Figure 1B**; see **Table S1**), as demonstrated previously (28, 41).

### 3.2 Taxa composition of the fruit bat gut microbiota

Altogether, 1,235 ASVs were identified from 72 anal samples of Egyptian fruit bats (six samples belonging to two bats were lost during the quality control stage, see ‘Sequence analysis and taxonomic identification’ for details). The final reads represented a total of 426 bacterial species, belonging mainly to the phyla Proteobacteria (mean relative abundance of 61% for challenged bats and 59% for control bats) and Firmicutes (mean relative abundance of 34% for challenged bats and 39% for control bats), as observed previously in this bat species (16).

### 3.3 Gut bacterial community dynamics of challenged bats

The GM composition of challenged bats significantly differed from that of control animals (PERMANOVA: treatment - F=1.733, Df=1, R^2 =^ 0.022, *p*=0.033, **Table 1**). This difference was significantly correlated with the haptoglobin concentration (PERMANOVA: haptoglobin - F=2.381, Df=1, R^2 =^ 0.032, *p*=0.006, treatment × haptoglobin: F=1.030, Df =1, R^2 =^ 0.014, *p*=0.408; **Table 1**), but more strongly with sampling time, as revealed by the PERMANOVA test (sampling time - F=2.566, Df =2, R^2 =^ 0.067, *p*=0.001, treatment × time: F=1.581, Df =2, R^2 =^ 0.041, *p*=0.031; **Table 1**).

**Table 1:** The effect of treatment (control *vs*. LPS), sampling time (0h, 24h, 48h) and haptoglobin concentration on the gut bacterial community composition of fruit bats.

	Df	F	R^2^	*p*
Treatment and sampling time				
**Treatment**	1	1.733	0.022	0.033*
**Time**	2	2.566	0.067	0.001**
**Treatment × Time**	2	1.581	0.041	0.031*
**Residuals**	66		0.868	
**Total**	71		1	
Treatment and Haptoglobin				
**Treatment**	1	1.666	0.022	0.035*
**Haptoglobin**	1	2.381	0.032	0.006**
**Treatment × Haptoglobin**	1	1.030	0.014	0.408
**Residuals**	68		0.930	
**Total**	71		1	

The permutational analysis of variance (PERMANOVA), i.e., ADONIS, is based on a Bray–Curtis dissimilarity matrix. Since ADONIS is sensitive to collinearity, sampling time and haptoglobin concentration (r=0.64) were tested separately. Bat identity was used as a nested factor. LPS, lipopolysaccharides. *p<0.05, **p<0.01.

**Figure 2:**
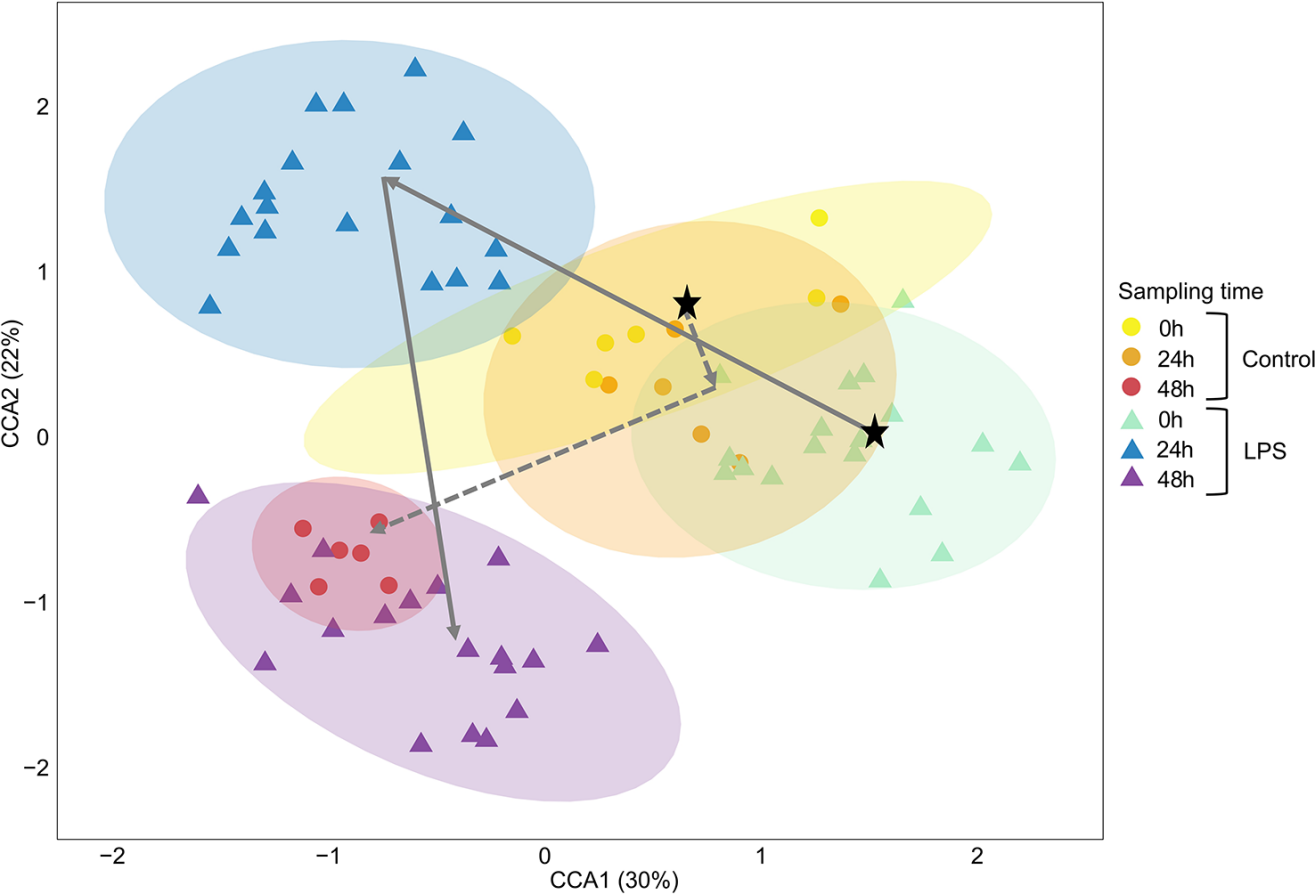
Canonical correspondence analysis (CCA) ordination plot of the relationships between the bat gut bacterial community composition, sampling time (0h, 24h, 48h) and treatment group (control *vs*. LPS). The CCA revealed that the gut bacterial community composition of bats clustered by sampling time at time points 0h (yellow and green) and 48h (red and purple) and by treatment at time 24h (orange and blue). The first and second canonical correspondence axes are shown. Values in brackets represent the percentage of total variance explained by the axis. Each dot represents a sample (*i.e.*, of an individual at a time point). The average direction of the temporal shift in the bacterial community composition is marked by dashed (control) or full (LPS) arrows with a black star depicting the starting point. LPS, lipopolysaccharides.

To further visualize the relationship between treatment and sampling time on the GM composition of fruit bats, a canonical correspondence analysis (CCA) was performed. The CCA ordination plot showed that the GM samples of challenged bats clearly separated by sampling time (CCA model: F=1.3415, *p*=0.001; **Figure 2**). A temporal shift was also evident in the control bats (mostly between the GM samples of times 0h-24h, and the samples of time 48h, **Figure 2**), in accordance with the previously documented coordinated temporal changes which occur in the gut microbiota of healthy individuals (16). The difference in the GM composition between control and challenged bats was most apparent 24h after the immune challenge (PERMANOVA: 24h - F=3.152, Df=1, R^2 =^ 0.125, *p*=0.001; **Figure 2**), whereas the GM was similar between the control and challenged bats prior the LPS injection (0h) and 48h post injection (PERMANOVA: 0h - F=0.732, Df=1, R^2 =^ 0.032, *p*=0.715, 48h - F=1.178, Df=1, R^2 =^ 0.051, *p*=0.265; **Figure 2**). Interestingly, the bats seemed to regain the colony’s group GM composition rapidly, within 48h of the LPS challenge.

In the CCA analysis, treatment and sampling time (constrained variables) accounted for 6.8% of the variance, while bat identity (conditional variable) accounted for 37% of the variance. The first two canonical axes accounted for 52% of the variation observed in the gut bacterial community composition (*i.e.*, 52% of the 6.8% explained variance were due to sampling time and treatment). About 55% of the variance was unexplained by the CCA model (**Table S2**).

Finally, the diversity (Shannon H’) and richness (Fisher alpha) of the GM composition were relatively similar between control and challenged bats at time 0h (ART ANOVA: Shannon H’ - F_1,21 =_ 0.791, *p*=0.383, Fisher alpha - F_1,21 =_ 0.521, *p*=0.478; **Tables S3, 4**) and showed little change over time, regardless of treatment (ART ANOVA: Shannon H’ - F_2,42 =_ 1.367, *p*=0.265, Fisher alpha - F_2,42 =_ 1.132, *p*=0.331; **Tables S3, 4**).

**Figure 3:**
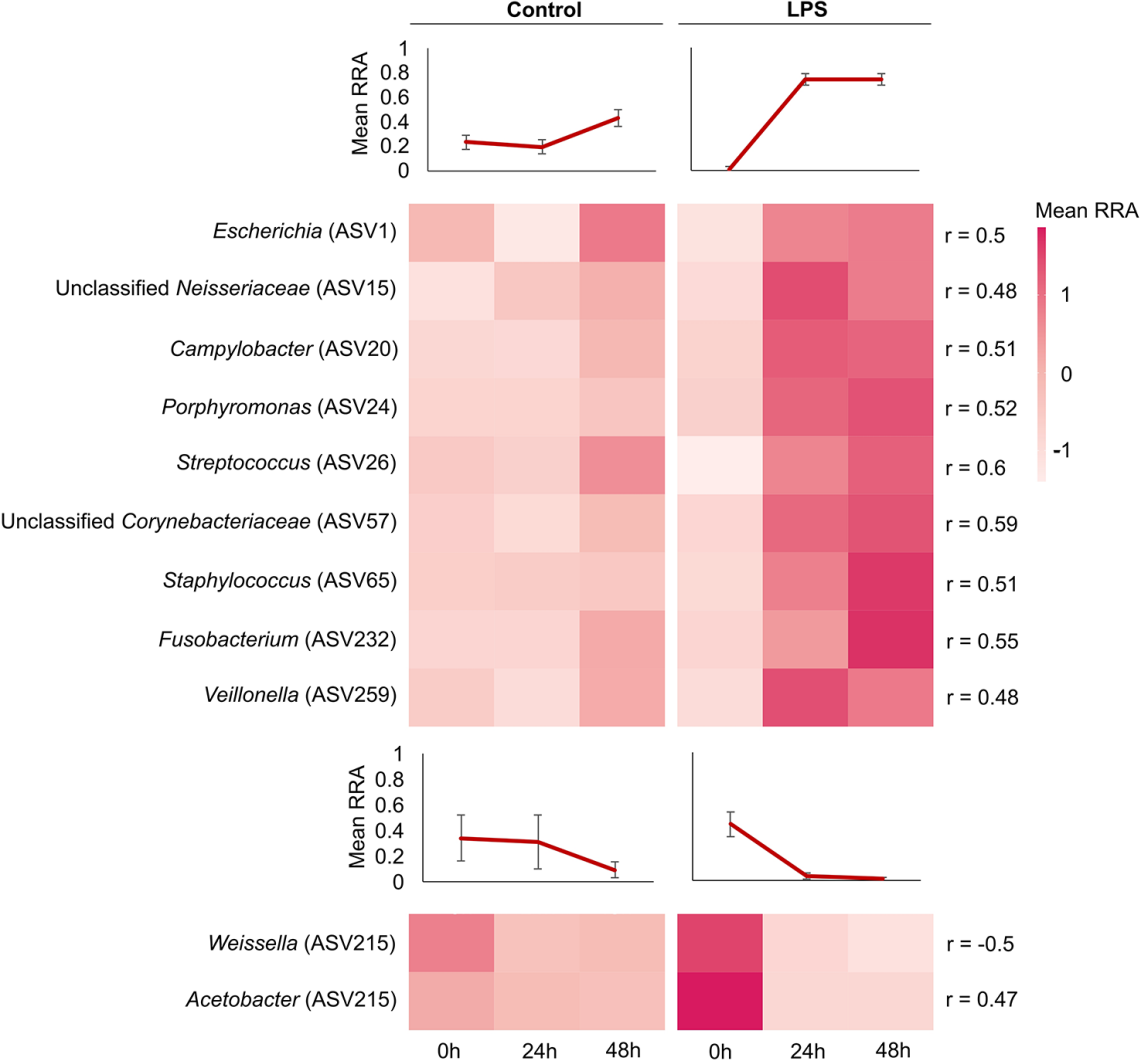
Bacterial ASVs that correlated with the strength of the immune response (haptoglobin concentration) in bats. Matrices show the relative read abundance (RRA) of amplicon sequence variants (ASVs) that correlated with haptoglobin concentrations, at different sampling time points. Only ASVs that presented significant Spearman r values (*p* adjust value < 0.05) with haptoglobin are presented and their r value is shown on the right. Positive correlations appear at the top of the graph and negative correlations appear at the bottom. The color scale represents the RRA of each ASV. Mean RRA values of ASVs in each time point (line graphs) are presented above each block (control bats – positive and negative correlations, challenged bats – positive and negative correlations). LPS, lipopolysaccharides. Each ASV genus affiliation (where possible) is denoted next to the ASV numbers on the left.

To investigate the specific changes in the GM composition of bats following the immune challenge, we performed a Spearman correlation test between ASVs and haptoglobin concentration of challenged bats (corrected with BY for multiple hypothesis testing; see Methods section). This test revealed 11 significant correlations (after correcting for multiple comparisons) - nine ASVs that were positively correlated with haptoglobin (*i.e.*, their relative abundance increased with the increase in haptoglobin concentration) and two which were negatively correlated with haptoglobin (*i.e.*, their relative abundance decreased with the increase in haptoglobin concentration; **Figure 3**). Positively correlated ASVs included the genera *Escherichia, Campylobacter, Porphyromonas, Streptococcus, Staphylococcus, Fusobacterium, Veillonella* and unclassified *Neisseriaceae* and *Corynebacteriaceae*, while negatively correlated ASVs included the genera *Weissella* and *Acetobacter* (**Figure 3**).

**Figure 4:**
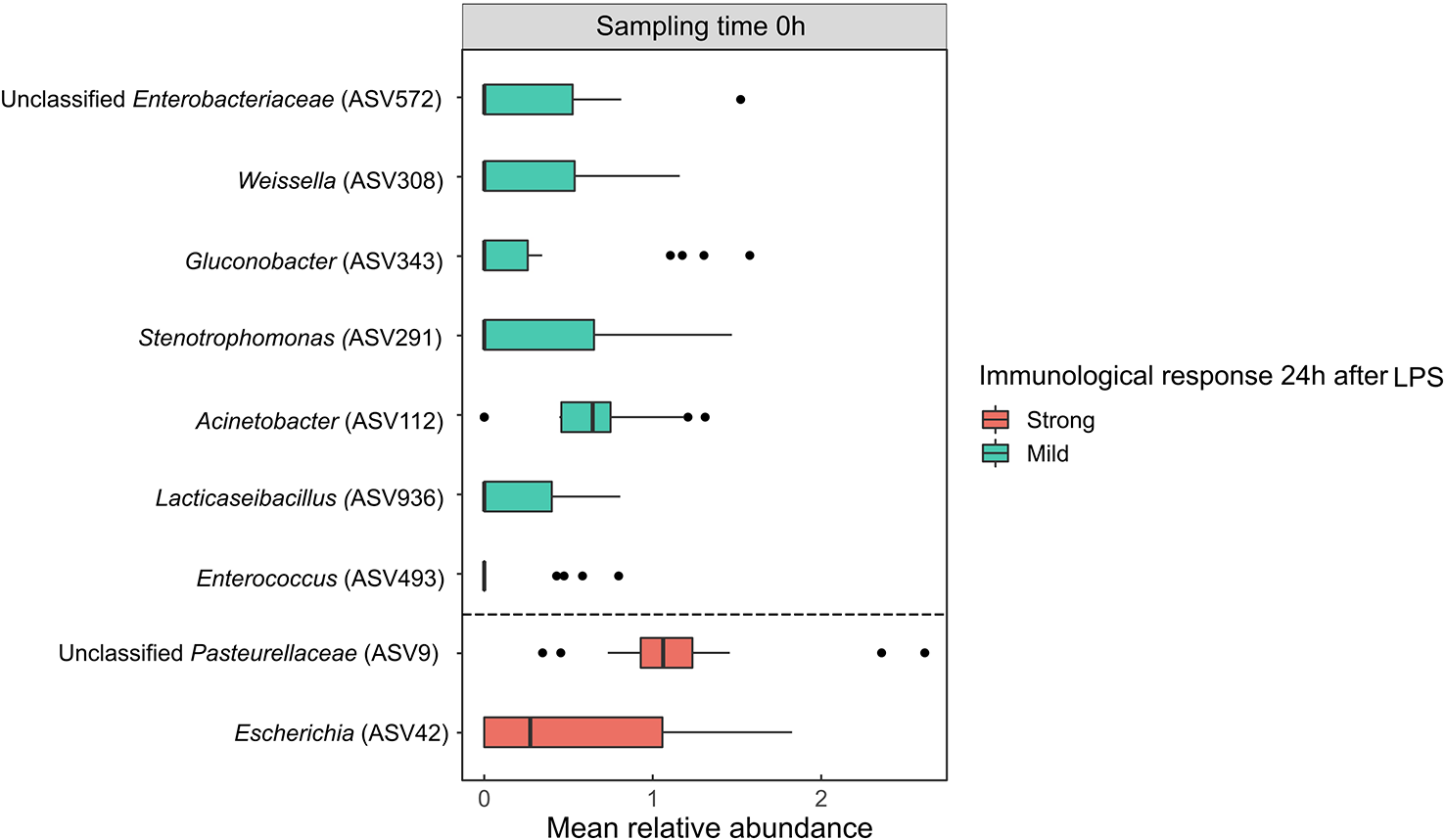
Bacterial amplicon sequence variants (ASVs) suggested by the linear discriminant analysis (LDA) effect size (LEfSe) analysis to be potential predictors of the strength of the immune response. ASVs which differed significantly between bats with mild and strong immune responses at time 0h, as confirmed by LEfSe (*p*<0.05, LDA score>2.0), are presented. The bars are colored according to bat haptoglobin production category 24h after the administration of the LPS challenge, and ASVs predicted to cause a strong immune response are separated from those predicted to cause a mild immune response by a dashed line. Results are presented as mean ± SEM. LPS, lipopolysaccharides.

To examine whether the immune response in bats depends on the composition of their GM prior to the exposure to an immune challenge, we checked which ASVs at time 0h were predictive of haptoglobin concentration at time 24h. To do so, an LDA effect size (LEfSe) analysis was performed on data of the challenged bats only, using read counts of ASVs at time 0h (*i.e.*, before the administration of LPS) against the haptoglobin concentration 24h after the administration of LPS (we divided the challenged bats into two groups according to their haptoglobin concentration – above or below the median; see Methods section). A total of nine ASVs were suggested by this analysis as potential predictors of the strength of the immune response (**Figures 4**, **S3**). Specifically, bats with high abundances of the genera *Enterococcus, Lacticaseibacillus, Acinetobacter, Stenotrophomonas, Gluconobacter, Weissella* and unclassified *Enterobacteriaceae* at time 0h showed reduced immune responses, while bats with a high abundance of the genera *Escherichia* and unclassified *Pasteurellaceae* at time 0h exhibited strong immune responses following the injection of LPS (**Figure 4**).

No overlap was observed at the ASV level between bacteria that correlated with haptoglobin concentration (**Figure 3**) and those suggested by the LEfSe analysis (**Figure 4**), yet an overlap was evident at the genus level - *Escherichia* and *Weissella*, were shared in both analyses, displaying opposite effects. *Escherichia*, which in high abundances at time 0h may predict the occurrence of a strong immune response, was positively correlated with haptoglobin. *Weissella*, which in high abundances at time 0h may predict the occurrence of a mild immune response (following an immune challenge), was negatively correlated with haptoglobin.

## 4 Discussion

In this study, we sought to explore GM dynamics in healthy and immune challenged Egyptian fruit bats, a frugivorous species that lives in densely populated colonies. We experimentally induced an acute phase response in the bats using LPS, which enabled us to examine the effect of a bacterial-like infection on GM diversity and structure. We revealed that the immune challenge causes a shift in the composition of the bat GM, which was especially evident 24h after the LPS injection. This shift was significantly correlated with haptoglobin concentration implying that physiological changes following immune responses correspond with changes in the GM. The overall GM shift due to the immune challenge was rapid, lasting 48 hours until the bats recovered and resumed foraging and social activities. The resilient nature of their GM may provide fruit bats with an adaptive advantage to cope with disease and maintain colony health.

### 4.1 Gut bacterial community dynamics of challenged bats

Challenged bats showed clear evidence of an acute phase response, such as body weight loss and an increased haptoglobin concentration (**Figure 1**). This inflammatory state was also evident in the GM of challenged bats, which was compositionally different from that of healthy control bats (especially 24h after the challenge, **Table 1** and **Figure 2**), suggesting that the challenge caused a shift in the GM. Indeed, several recent studies demonstrated altered GM compositions in diseased and/or immune-challenged animals, including Atlantic salmon (42), tilapia (43), rhesus macaques (44) and barn swallows (45). While some of these studies demonstrated reduced alpha diversity in the GM of sick animals (*i.e (* 43 *)*), others demonstrated no effect on alpha diversity at all (*i.e (* 44, 45 *)*), as observed in this study (alpha diversity was similar between control and challenged bats; **Tables S3, 4**). This likely occurred due to the fact that some bacterial sequences changed only in abundance, while others were replaced, resulting in a distinct composition, but an overall similar alpha diversity. While Shannon indices of both control and challenged bats were lower compared to the findings of our previous study on the GM of Egyptian fruit bats (16), they were consistent with the values observed generally in the GM of bats (46).

A comparison between the GM of control and challenged bats revealed several ASVs which significantly correlated with haptoglobin concentration (**Figure 3**). Control bats, displaying a low concentration of haptoglobin, were characterized by higher relative abundances of bacteria of the genera *Weissella* and *Acetobacter* (**Figure 3**). Interestingly, species of these genera are found on various fruits (and nectar), which are the main food source of Egyptian fruit bats (47). Indeed, diet is considered the most important factor shaping the GM of many organisms [*e.g (* 19, 48–50 *)*]. While *Weissella* and *Acetobacter* have been previously identified in bats with different diets (18, 51, 52), their importance in host health remains unknown. *Weissella* belongs to the lactic acid bacteria (family: *Lactobacillaceae*) which contains many species with probiotic and prebitoic properties, and antimicrobial activity (53). Although rare, some species may be pathogenic (54). The genus *Acetobacter* belongs to the acetic acid bacteria (family: *Acetobacteraceae*) which are vastly used in the food production industry. Like *Weissella*, some species of this genus have probiotic characteristics (55, 56). The fact that both genera were negatively correlated with haptoglobin concentration (**Figure 3**) and that high abundances of *Weissella* prior infection may predict the occurrence of a mild immune response (**Figure 4**) suggests that these genera might be important in providing bats with protection against pathogens. This could be experimentally tested by administering *Weissella* to bats prior an LPS challenge and examining the effect on the strength of the immune response. Unsurprisingly challenged bats, displaying increased haptoglobin concentrations, were characterized by higher relative abundances of bacteria of the genera *Escherichia, Campylobacter, Porphyromonas, Streptococcus, Staphylococcus, Fusobacterium, Veillonella* and the families *Neisseriaceae* and *Corynebacteriaceae* (**Figure 3**), all of which include commensal species with the potential to be opportunistic pathogens in humans and other vertebrates (57–62). Similarly, other animals, such as birds, fishes and monkeys, exhibit increased abundances of opportunistic commensal species following infection. Like bats, immune-challenged barn swallows showed increased abundances of *Staphylococcus* and *Streptococcus* (but also of *Bacillus* and *Dysgonomonas*) in their gut, while healthy individuals had increased abundances of *Enterococcus* and *Lactococcus* (45). Atlantic salmon, suffering external bacterial skin infections, exhibited increased abundances of *Aliivibrio* in their gut, while healthy fish exhibited increased abundances of *Mycoplasma* (42). Diseased tilapia displayed high abundances of *Vibrio* in their intestine compared to *Cetobacterium* in healthy fish (43). The GM of rhesus macaques infected with tuberculosis were enriched with the bacterial families *Lachnospiraceae* and *Clostridiaceae* (44). Likewise, the GM of humans suffering from disease (such as diabetes and colon cancer) also exhibits reduced abundances of beneficial bacteria and enrichment of potential pathogens (63). An acute phase response is the costliest part of the immune response, both in term of energy and pathology (*e.g*., immunopathology). A shift in the GM composition, especially an increase in the abundance of opportunistic bacterial pathogens, might be another cost associated with activation of this immune branch.

The LEfSe analysis revealed that the baseline GM (*i.e.*, prior immune challenge) of bats that developed a strong immune response was distinct from that of bats which developed a mild immune response. Specifically, ASVs belonging to the genera *Escherichia* and the family *Pasteurellaceae* were enriched in the GM of bats which developed a strong immune response (**Figures 4**, **S3**). Indeed these bacteria include several species known as opportunistic invaders (58, 62) which can make their hosts more susceptible to disease in high abundances (64). The strong immune response in individuals that exhibited increased abundances of *Escherichia* and unclassified *Pasteurellaceae* may be related to the fact that these bacteria also contain LPS in their outer membranes. Although these LPS antigens can be different from the one we used, it has been shown that *E.coli* infections and LPS challenges can prime neutrophils and antibody responses towards both homologous and heterologous antigens (65). However, other Gram-negative bacteria exhibited increased abundances prior to the immune challenge (e.g., *Acetobacter* and *Tatumella*) and were not found as predictors of the strength of the immune response, implying that a primed response is not necessarily due to the previous LPS exposure. Nonetheless, LPS can be a stimulant of host immunity (66), and thus future research should consider conducting similar experiments with other bacterial antigens, especially with LPS from other species which are not part of the GM (*e.g.*, *Salmonella*). *Enterococcus, Lacticaseibacillus*, and *Weissella* on the other hand, which were enriched in the GM of bats that developed a mild immune response (**Figures 4**, **S3**), belong to the lactic acid bacteria. This group contains many species with probiotic traits (53), which may protect their host against infections. Our findings suggest these baseline microbial communities may be important predictors of the strength of an immune response (and implicit infection severity), determining if the host is more susceptible to infection or not. Whether the severity of infection is due to the GM effect on host resistance *via* immunity function paths or vice versa, remains to be explored.

The composition of the GM of both control and challenged bats changed significantly over time (**Table 1**, **Figure 2**), as previously observed in this species (16) and others (67). Such a temporal shift in the GM may be explained by external factors, such as food availability and seasonal changes (although we controlled for these changes in the experiment), and internal factors, such as physiological state and ecological succession. Different physiological states, such as reproduction and stress, are also known to affect the GM composition (22, 68). Since the bats in our study (males only) were acclimated to captivity conditions and handling, we assume that stress and reproduction state are not the reasons for the compositional changes in the GM of bats. Rather this temporal shift may be an adaptive trait, enabling bats, as a colony, to rapidly adjust to varying food sources and environmental changes.

### 4.2 The gut microbiota of fruit bats shows high resilience to an immune challenge

Despite exhibiting compositional changes following the immune challenge, the bat GM showed high resilience, regaining the colony’s group GM composition within 48h of the challenge. Furthermore, both diversity and richness (alpha diversity) of the GM remained unchanged following the immune challenge, highlighting the resilience of the bat GM. These findings correspond with our recent study in which we demonstrated that challenged bats choose to remain isolated for two days, resuming foraging trips (*i.e.*, feeding) and social interactions 48-72h post-recovery from an immune response provoked by a bacterial endotoxin, even though haptoglobin concentration is still high (28). Resuming these activities likely facilitates stabilizing the GM – bats suffering from anorexia and extreme lethargy resume feeding and with it the supply of carbohydrates and beneficial bacteria (*Weissella* and *Acetobacter* originating in fruit) to the GM. Moreover, self-isolated bats return to their densely populated, high-contact groups, resuming social interactions such as licking themselves and one another, possibly re-introducing bacteria into the gut (16). Indeed, examining the trends of the 11 ASVs that correlated with the haptoglobin concentration (**Figure 3**) suggests that after 48h the GM of control bats (n=6) became more similar to that of the challenged bats (n=18). This is reasonable in light of the well documented microbiota sharing in this species (16, 69). In other words, we suggest that the GM of challenged bats (which changed due to the LPS challenge) influenced that of control bats, likely *via* social interactions. In nature the shift in the gut bacterial community due to microbiota sharing will probably be in the direction of the healthy colony GM (*i.e.*, majority of individuals) rather than the GM of sick individuals, as seen in this study. The bats adaptation to physiological changes during flight may also partially explain the GM resilience - their body temperature increases in flight within minutes by 3-5°C (70), thus bats are used to dealing with elevated body temperatures as those which occur during infection. Lack of stability in the GM (dysbiosis) is often associated with disease (71). Thus, resuming the balance of the GM following infection (*i.e.*, high resilience) is likely critical for bat health (72), especially since it is essential to food absorption (14), social recognition (15, 16), and pathogen defense (17). Living in dense groups may facilitate the stabilization of the GM in infected bats, and possibly in other social species roosting in close proximity, providing them with an adaptive advantage to cope with disease and maintain colony health.

### 4.3 Conclusion

In this study we show that an immune challenge associated with a bacterial endotoxin causes a shift in the composition of the fruit bat GM, which is correlated with the concentration of the acute phase protein haptoglobin, highlighting the association between immune responses and GM structure. Despite these compositional changes, the bat GM showed high resilience, restabilizing within 48h of the immune challenge, as bats resumed foraging and social interactions. Not all bats responded equally to the immune challenge – some developed a strong immune response while others developed a mild response – which changed according to their baseline GM (prior to the LPS injection). This finding suggests that baseline microbial communities may be important predictors for the strength of an immune response, and implicit of infection severity. Our findings emphasize the importance of integrating microbial ecology in ecoimmunological studies of wild populations.

## Data availability statement

The datasets presented in this study can be found in online repositories. The names of the repository/repositories and accession number(s) can be found in the article/**Supplementary Material**.

## Ethics statement

The animal study was reviewed and approved by Animal care and experimental procedures were approved by Tel AvivUniversity IACUC under approval form ID 04-19-002.

## Author contributions

Conceived and designed the experiments: MW, KM, GC and YY. Performed the experiments: MW and KM. Analyzed the data (including bioinformatic analysis of RAW data) and prepared the figures: TB. Wrote the first draft: TB. All authors contributed to the article and approved the submitted version.
