# Supplementary material for: In sickness and in health: the dynamics of the fruit bat gut microbiota under a bacterial antigen challenge and its association with the immune response
Source: Front Immunol. 2023 Apr 11;14:1152107. doi: 10.3389/fimmu.2023.1152107 (PMC10126333; doi:10.3389/fimmu.2023.1152107)
Supplement: Supplementary file 8 [file DataSheet_8.docx]

Supplementary Material

**In sickness and in health: The dynamics of the fruit bat gut microbiota under a bacterial antigen challenge and its association with the immune response**

**Tali S. Berman^1^***^†^**, Maya Weinberg^1^***^†^**, Kelsey R. Moreno^1^**^ɤ^**, Gábor Á. Czirják^2^, Yossi Yovel^1,3^**

^1^Department of Zoology, Tel Aviv University, Tel Aviv – Yafo 6997801, Israel

^2^Department of Wildlife Diseases, Leibniz Institute for Zoo and Wildlife Research, Berlin 10315, Germany

^3^Sagol School of Neuroscience, Tel Aviv University, Tel Aviv – Yafo 6997801, Israel

*** Correspondence:**Tali S. Berman, [talisberman@gmail.com](mailto:talisberman@gmail.com)

Maya Weinberg, [mayababa@gmail.com](mailto:mayababa@gmail.com)

**Current affiliation:**

^ɤ^Kelsey R. Moreno, Department of Psychology, Saint Xavier University, Chicago, USA

^†^**These authors contributed equally to this work**

# Supplementary Figures


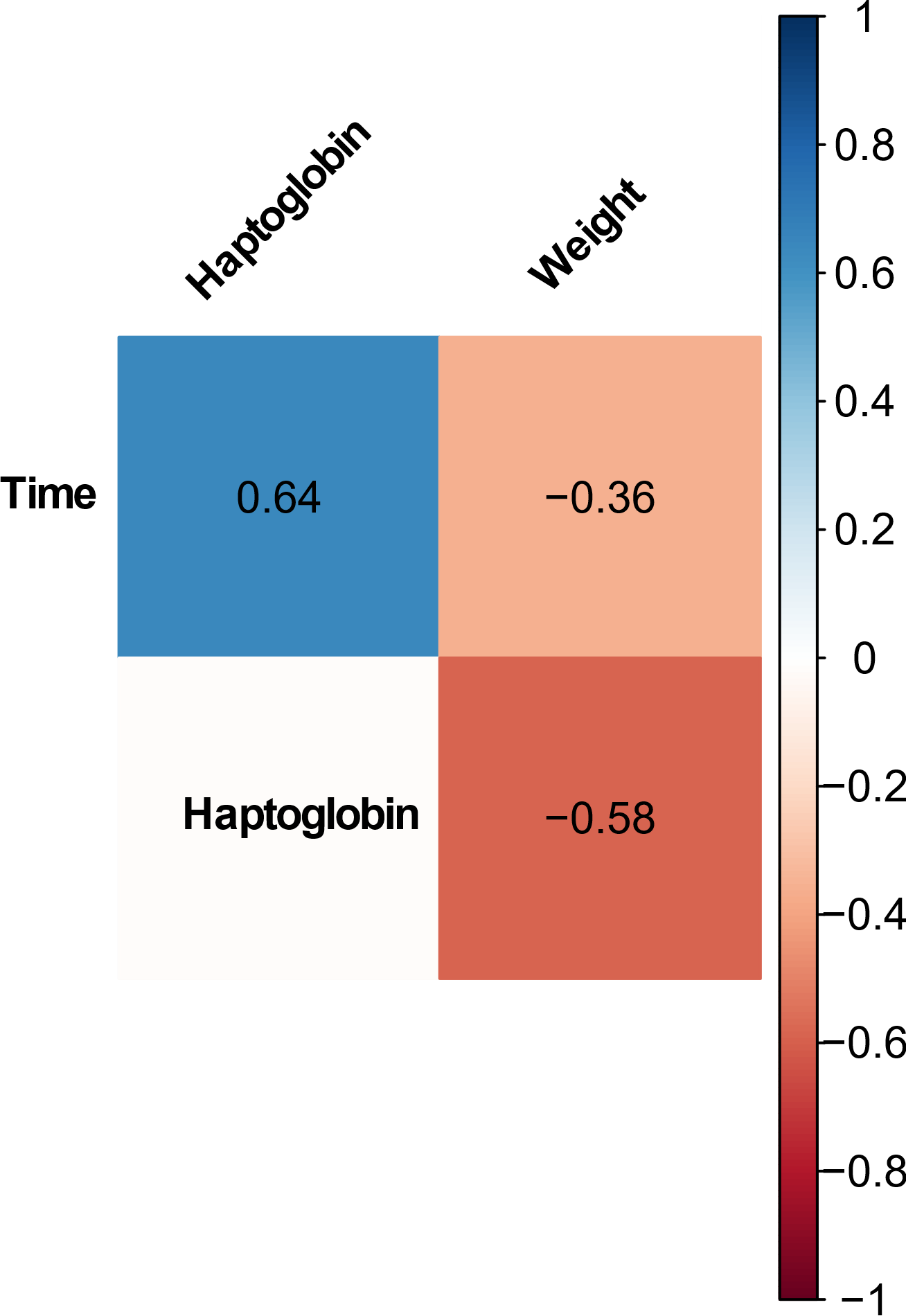


**Fig. S1. Visualization of the correlation matrix between the metadata variables haptoglobin, weight and sampling time.** Blue indicates a positive correlation and red indicated a negative correlation. Numbers in the boxes represent r values. Only significant correlations (*p*<0.05) appear in the plot.


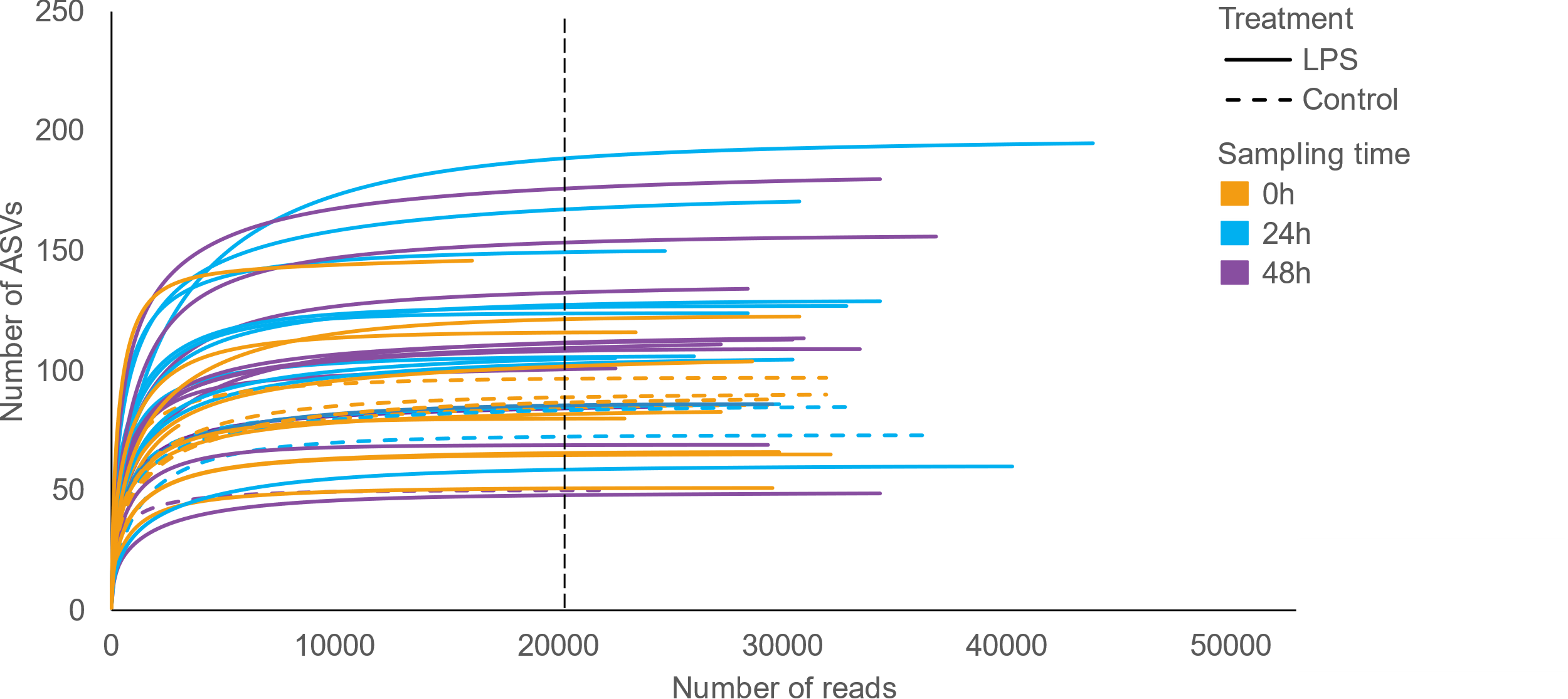


**Fig. S2. Rarefaction curve of control and treatment (LPS) samples from the different sampling times (pre-injection, 24h and 48h post-injection).** The rarefaction curves of most of the samples reached an asymptote level suggesting that our sampling effort was sufficient to obtain a full estimate of ASV richness. The data was rarefied to a minimum sequence depth of 20,000 sequences per sample (marked by dashed line).

**
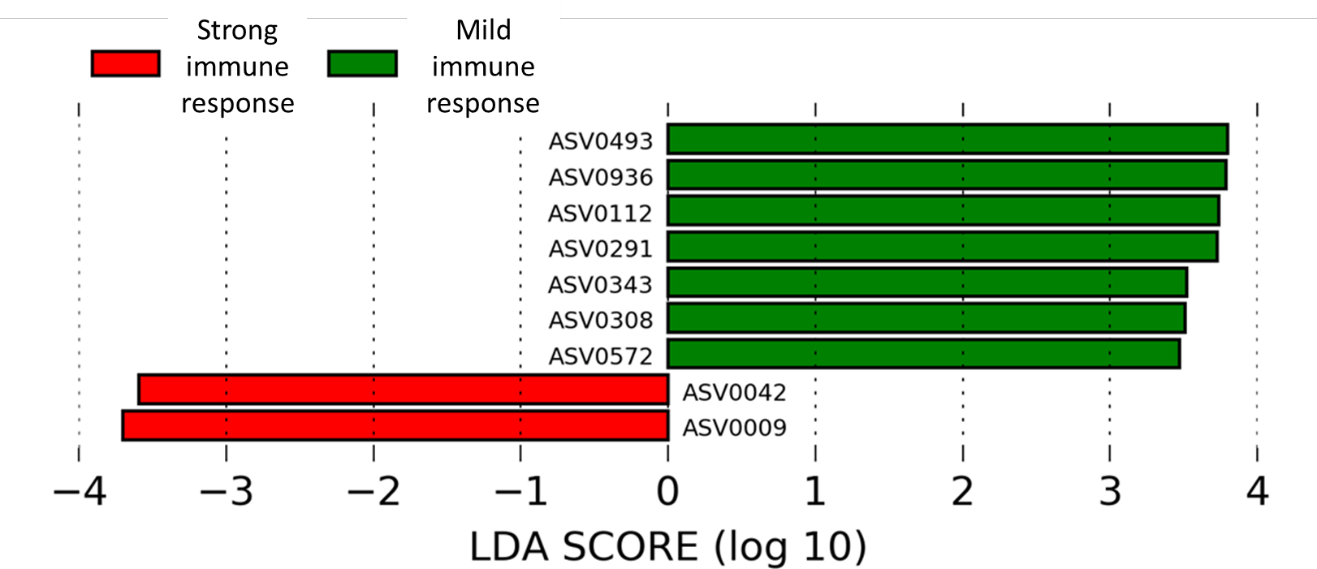
**

**Fig. S3. LDA scores of ASVs** **with significant differential abundances at time 0h (prior LPS injection) between bats who developed a mild and strong** **immune response.** The amplicon sequence variants (ASVs) presented are those with the highest LDA score by LEfSe analysis. Negative LDA scores (left) are enriched in bats with a strong immune response, whereas positive LDA scores (right) are enriched in bats with a mild immune response.

# Supplementary tables

**Table S1. Aligned Rank Transform (ART) ANOVA table examining differences in haptoglobin levels and weight between bats of the control and treatment (LPS) groups during three sampling times.** Haptoglobin data showed no homogeneity of variance (F test: Haptoglobin - F= 0.08, *p*=<0.001). None of the data presented a normal distribution (Shapiro Wilk test: Haptoglobin – W=0.877, *p*<0.001; Weight - W=0.938, *p*=0.001), thus an ART ANOVA was performed.

| Immune response | Effect | Statistic values | | |
| --- | --- | --- | --- | --- |
|  |  | **F** | **Df** | ***p*** |
| Haptoglobin | Treatment (PBS *vs*. LPS) | 35.762 | 1,22 | **<0.001** |
|  | Time (0h *vs*. 24h *vs*. 48h) | 162.248 | 1,44 | **<0.001** |
|  | Treatment × Time | 78.773 | 1,44 | **<0.001** |
|  | Treatment (PBS *vs*. LPS) | 4.515 | 1,22 | **0.045** |
| Weight | Time (0h *vs*. 24h *vs*. 48h) | 19.828 | 1,44 | **<0.001** |
|  | Treatment × Time | 9.827 | 1,44 | **<0.001** |

**Table S2. Canonical correspondence analysis (CCA) results for the relationships between bat gut bacterial community composition, bat identity, sampling time (0h, 24h, 48h) and treatment group (control *vs*. LPS).** Bat identity was set as a conditional variable. Constrained variables (treatment and sampling time) accounted for 6.8% of the variance in the CCA. Significance of the CCA model was tested using a permutation test (anova.cca). The CCA model was significant (F = 1.3415, *p* = 0.001).

|  | **Inertia** | **Proportion** | **Rank** |
| --- | --- | --- | --- |
| **Total** | 12.331 | 1 |  |
| **Conditional** | 4.602 | 0.373 | 23 |
| **Constrained** | 0.840 | 0.068 | 4 |
| **Unconstrained** | 6.889 | 0.558 | 44 |

**Table S3. Diversity (Shannon H’) and richness (Fisher alpha) indices between bats of the control and treatment (LPS) groups during three sampling times.** Diversity and richness of the gut bacterial community composition were similar between control and challenged bats and showed little change over time regardless of treatment. For full statistics see Table S4 below.

| Treatment | Time | | |
| --- | --- | --- | --- |
|  | 0h | 24h | 48h |
| Shannon H’ | | | |
| Control | 1.915 ± 0.295 | 1.944 ± 0.204 | 2.374 ± 0.325 |
| LPS | 2.645 ± 0.191 | 2.183 ± 0.200 | 2.189 ± 0.153 |
| Fisher alpha | | | |
| Control | 11.514 ± 1.044 | 10.310 ± 1.780 | 14.391 ± 1.912 |
| LPS | 15.248 ± 2.163 | 13.214 ± 1.339 | 14.712 ± 1.637 |

**Table S4. Aligned Rank Transform (ART) ANOVA table examining differences in diversity (Shannon H’) and richness (Fisher alpha) indices between bats of the control and treatment (LPS) groups during three sampling times.** No significant differences were observed in diversity or richness of the gut bacterial community composition between control and challenged bats during any of the sampling times.

| Immune response | Effect | Statistic values | | |
| --- | --- | --- | --- | --- |
|  |  | **F** | **Df** | ***p*** |
| Shannon H’ | Treatment (PBS *vs*. LPS) | 0.791 | 1,21 | 0.383 |
|  | Time (0h *vs*. 24h *vs*. 48h) | 1.367 | 2,42 | 0.265 |
|  | Treatment × Time | 1.562 | 2,42 | 0.221 |
| Fisher alpha | Treatment (PBS *vs*. LPS) | 0.521 | 1,21 | 0.478 |
|  | Time (0h *vs*. 24h *vs*. 48h) | 1.132 | 2,42 | 0.331 |
|  | Treatment × Time | 0.588 | 2,42 | 0.559 |
